# Aging-related alternative splicing drive neoantigen emergence revealed by transcriptome analysis of 1,255 human blood samples

**DOI:** 10.3389/fragi.2025.1575862

**Published:** 2025-05-09

**Authors:** Shuhan Li, Haohao Lv, Renxin Zhang, Jinjun Li, Zhiyuan Chen, Naixue Yang, Shaoxing Dai

**Affiliations:** ^1^ State Key Laboratory of Primate Biomedical Research, Institute of Primate Translational Medicine, Kunming University of Science and Technology, Kunming, Yunnan, China; ^2^ Yunnan Key Laboratory of Primate Biomedical Research, Kunming, Yunnan, China

**Keywords:** aging, transcriptome analysis, alternative splicing, immunosenescence, neoantigens, anti-aging immunotherapy

## Abstract

This study aimed to identify age-related genes and alternative splicing (AS) events by comprehensive transcriptome analysis of 1,255 healthy blood samples from individuals aged 8–87 years. We identified 1,029 up-regulated and 1,186 down-regulated genes in older individuals, including 17 genes overlapped with known aging-associated genes, such as TFAP2A and Klotho. Gene set enrichment analysis revealed significant alterations in immunoregulatory and metabolic pathways during aging. However, many senescence-associated secretory phenotypes (SASP) involved genes did not exhibit changes in gene expression, suggesting that AS events may reveal additional age-related mechanisms. Aging also altered 6,320 AS events in 4,566 genes, impacting immune-related protein domains. The RNA-binding protein RBMS3 emerged as a key regulator of aging-specific AS events. In addition, neoantigen prediction analyses further identified potential neoantigens generated by aging-related AS events, with the HLA-C14:02 allele presenting the most neoantigenic peptides. Notably, 60 neoantigenic peptides were confirmed using proteomic data from elderly individuals, suggesting their potential as novel targets for anti-aging immunotherapy. Our study provides new insights into the role of alternative splicing in aging, highlights promising avenues for anti-aging immunotherapy.

## Introduction

Aging is a complex biological process marked by progressive dysfunction of tissues and organs, increased susceptibility to mortality, and pervasive effects on nearly all physiological systems. Key hallmarks of aging include genomic instability, telomere shortening, epigenetic alterations, protein homeostasis imbalance, mitochondrial dysfunction, stem cell depletion, and altered cellular communication (Rando and Wyss-Coray, 2021; Dodig et al., 2019; López-Otín et al., 2013). These molecular changes ultimately lead to impaired tissue function and a significantly higher risk of diseases such as cancer and cardiovascular disease, particularly after age 60 (Khosla et al., 2020; Melzer et al., 2020; Lehallier et al., 2019; Campisi et al., 2019; Oliynyk, 2019). Current treatments for aging-related diseases are often focused on individual conditions. However, this approach may not address the connections between different diseases, limiting its effectiveness in extending a healthy lifespan. A deeper understanding of the overall mechanisms of aging is crucial for developing better treatments (Ben-Hur et al., 2013).

Studies have shown that blood contains factors that both promote aging and support rejuvenation (Bieri et al., 2023). Using heterochronic xenobiotic experiments in which blood from older animals was delivered to younger animals at different time points, multiple phenotypes of accelerated aging were observed in younger recipients (Villeda et al., 2011; Katsimpardi et al., 2014; Rebo et al., 2016). Moreover, AS is a crucial aspect of eukaryotic gene expression, and significant changes in splicing patterns have been observed during aging and senescence (Deschênes and Chabot, 2017). For example, mutations affecting splicing in the *LMNA* gene, which encodes lamin A/C, are known to cause Hutchinson-Gilford progeria syndrome, a condition characterized by premature aging in mice and human (Rodríguez et al., 2016). In neurodegenerative diseases such as Alzheimer’s disease (AD), aberrant splicing of susceptibility alleles in genes like *PICALM, CLU*, and *PTK2B* has been implicated in disease progression, further demonstrating the critical role of splicing regulation in age-related disorders (Raj et al., 2018). RNA-binding proteins (RBPs), which regulate splicing events, display dynamic expression changes in senescent tissues, and many of them are closely linked to the cellular senescence process. For example, SRSF1, QKI, and RBFOX2 are known to be down-regulated in senescent human fibroblasts, affecting global splicing fidelity and senescence-associated phenotypes (Holly et al., 2013; Blanco et al., 2008). However, large-scale transcriptomic investigations of aging-specific splicing patterns in human blood remain limited, leaving critical knowledge gaps regarding how AS contributes to immunosenescence and age-related antigenic remodeling.

Aging is accompanied by chronic inflammation, cellular senescence, and immune decline, creating a vicious cycle between inflammation and aging. Therefore, controlling inflammation may be a key strategy for anti-aging interventions (Li X. et al., 2023). As early as 1969, Walford introduced the concept of “immunological theory of aging,” suggesting that immune senescence reduces the body’s ability to fight tumors and eliminate senescent cells (Walford, 1964). In oncology, abnormal AS has been found to produce new antigens that are recognized by the immune system and trigger immune responses (Cheng et al., 2021). For instance, mutations in genes regulating RNA splicing are common in hematological malignancies, and intron retention (IR) has been identified as a potential source of tumor neoantigens in multiple myeloma (MM) patients (Dong et al., 2021). Neoantigens generated from aberrant splicing events may be presented on the cell surface via MHC molecules. These unique peptides, being immunologically novel, can serve as specific targets for engineered T cells in CAR-T therapy (Frankiw et al., 2019). Similarly, by studying age-specific splicing patterns, we may discover new antigenic epitopes that could serve as targets for anti-aging immunotherapy. NKG2D-CAR T cells have demonstrated the ability to effectively eliminate senescent cells in mouse and non-human primate (NHP) models without inducing significant adverse effects (Yang et al., 2023). Although CAR T-cell therapy has shown promise in aging-related disease models (Amor et al., 2020), there is still limited research on aging-specific antigenic epitopes, especially those derived from alternative splicing events. Importantly, clinical application of AS-derived neoantigens in CAR-T or other immunotherapies must proceed with caution, as they may trigger autoimmune responses if not properly filtered for specificity. A key challenge lies in identifying antigens that are uniquely expressed on deleterious senescent cells (Wu et al., 2024), minimizing the risk of off-target effects. Therefore, the discovery of reliable, selective neoantigens is a critical first step. In this study, we systematically analyzed transcriptomic data from 1,255 healthy human blood samples to reveal aging-specific splicing patterns. Aging was found to alter 6,320 AS events in 4,566 genes, significantly affecting immune-related protein domains. Neoantigen prediction analyses further identified potential neoantigens derived from aging-related AS events, with the HLA-C14:02 allele presenting the most neoantigenic peptides. Furthermore, the splicing factor RBMS3 emerged as a potential key regulator of aging-related splicing mechanisms. Importantly, a total of 60 predicted neoantigenic peptides were validated using proteomic data from elderly individuals, highlighting their therapeutic potential. These results not only deepen our understanding of age-associated transcriptomic changes but also provide a strong foundation for advancing anti-aging immunotherapy strategies.

## Methods

### Data acquisition

In this study, we aimed to identify age-related transcriptomic changes by applying stringent sample selection criteria: all included samples required detailed age records, confirmation of healthy donor status, and access to raw, unprocessed sequencing data. We programmatically compiled sample metadata for all human blood transcriptome datasets available in the GEO database and then applied these criteria to identify eligible datasets. Ultimately, 21 datasets comprising 1,255 human blood samples were selected for downstream analysis. RNA sequencing data were downloaded using wget or axel tools. Detailed information on the samples is provided in Supplementary Table S1. Additionally, we collected 307 aging-related genes from the GenAge database (Supplementary Table S2), a core component of the Human Aging Genomic Resources (HAGR), which contains extensive data on genes closely associated with aging processes. To further investigate immune-related changes during aging, we also gathered senescence-associated secretory phenotype (SASP) (Zhang et al., 2021), immune gene sets (Yoshihara et al., 2013), and inflammatory factor gene sets (Subramanian et al., 2005) from published literature (Supplementary Table S2). To validate the presence of predicted neoantigens in old individuals, we retrieved proteomic data from three blood sample datasets (PXD034030, PXD033493, PXD050061; detailed in Supplementary Table S3 **)** from the ProteomeXchange database, a global repository for proteomics data (Vizcaíno et al., 2014).

### RNA-seq data processing

First, quality control and filtering of the downloaded fastq data was performed using fastp (v0.22.0) (Chen et al., 2018). Sequencing reads with junctions, excessive undetermined bases (N), or low quality scores were removed. The following parameters were applied to optimize the process: window size was set to 16 (-w 16), minimum quality threshold was set to 25 (-q 25), minimum read length was set to 45 (-length_required 45), and a maximum of five unidentified bases were allowed (-n_base_limit 5). After filtering, the data were aligned to the reference human genome (GRCh38) downloaded from the GENCODE project. The genome index was generated using HISAT2 (v2.1.0) (Kim et al., 2019), and each sample was individually aligned to generate BAM files. The BAM files were then converted to SAM files using SAMtools (v1.5) (Li et al., 2009). Transcript assembly and quantification of gene and transcript expression levels were performed using StringTie (v2.1.2) (Pertea et al., 2015). Finally, to quantify gene expression across all samples, featureCounts (v2.0.3) (Liao et al., 2014) was used for gene-level quantification.

### Principal component analysis, gene expression analysis, enrichment analysis

To account for potential batch effects arising from the use of multiple GEO datasets, we first applied the removeBatchEffect function (Ritchie et al., 2015) in the limma package, incorporating both dataset source and sex as covariates. Differentially expressed genes were then identified using the limma package (Smyth, 2004), with significance thresholds set at p-value < 0.05 and |log2FC| > 1. To further explore the biological significance of these differentially expressed genes, we employed Gene Set Enrichment Analysis (GSEA) to identify significantly upregulated and down-regulated biological processes (upregulation: p-value < 0.05, |NES| > 1; down-regulation: p-value < 0.05, NES < −1). All results were visualized using ggplot2. Additionally, we used the gene set scoring function in GSEA to score samples from juvenile and elderly groups based on senescence-associated secretory phenotype (SASP), immune response, and inflammation-related gene sets. Violin plots were generated using the ggpubr package to illustrate these scores. Venn diagrams were used to illustrate the overlap between gene sets.

### Alternative splicing analysis

In this study, AS analysis was conducted on BAM files from the old and young sample groups using rMATS (v4.1.2) (Shen et al., 2014) to identify differential splicing events between the two groups. A junction counting (JC) approach was applied to quantify each AS event by targeting read and splice counts. All AS events were rigorously screened based on the criteria of absolute inclusion level difference (deltaPSI > 0.1) and false discovery rate (FDR < 0.05) between the old and young groups. Significant differential splicing events were visualized using volcano plots. The chromosomal distribution of differential AS events was determined based on genomic coordinates provided in the rMATS output files. Additionally, the overlap of these splicing changes was calculated and visualized using the upsetR package.

For genes associated with significant differential splicing events, we first performed protein-protein interaction (PPI) network analysis using the STRING database (Wagner and Fischer, 1974) to identify key network nodes and functional modules. The PPI network data were then imported into Cytoscape for further topology analysis and visualization editing. We then applied the rMATS output to rMAPS2 (version 2.0.0) (Hwang et al., 2020) for binding motif enrichment analysis. The analysis focused on the binding motifs of 115 known RNA-binding proteins, with significantly spliced regions serving as the target regions and non-significantly spliced regions used to estimate background binding levels. For motif enrichment analysis, we set 250 base pairs (bp) and 50 bp as the lengths for intronic and exonic regions, respectively, to examine and map the distribution of motifs associated with splicing events. In addition, ordinary least squares (OLS) linear regression was used to calculate the correlation between Percent Spliced In (PSI) values and the expression levels of genes with differential splicing events. Venn diagrams were used to depict the overlap between the senescence-associated secretory phenotype (SASP), immune gene set, and inflammatory factor gene set. The top 200 genes associated with significantly different splicing events were selected for Gene Ontology (GO) enrichment analysis using the clusterProfiler package (Wu et al., 2021).

### Neopeptides prediction

We obtained unspliced isoforms using chromosomal position information from the reference genome (GRCh38), including chromosome numbers and the start and stop base pair positions of each gene. Based on the rMATS analysis results, we deleted, added, or modified exons of the unspliced transcripts to construct spliced isoforms. Python scripts were used to compare the coding sequences (CDS) of spliced and unspliced isoforms for each differential splicing event.

Next, we applied InterProScan (Jones et al., 2014) to search for protein structural domains in the Pfam database and used R scripts to identify splicing events that overlapped with these domains. Using the translate function, we translated unspliced and spliced transcripts into amino acid sequences, which were divided into 9-amino acid peptides using a sliding-window technique (moving one residue at a time).

Additionally, we constructed peptide libraries using gffread (v0.11.4) (Pertea and Pertea, 2020), by translating transcripts quantified by StringTie (Pertea et al., 2015) from young and middle-aged samples into amino acid sequences, which were also split into 9-mer peptides. By excluding peptides from the spliced isoforms that matched those in the unspliced isoforms and in the young and middle-aged peptide libraries, we identified novel peptides specific to the aging process.

Finally, we used the OptiType tool (Szolek et al., 2014) for HLA typing of elderly individuals and combined it with NetMHCpan-4.1 (Reynisson et al., 2020) to predict the binding affinity of the novel peptides to HLA molecules. Peptides with a binding score below the 5% threshold were selected as potential neoantigen candidates. Using makeblastdb, we constructed a custom amino acid database from all peptide sequences derived from the mass spectrometry data of three elderly blood proteomic datasets (PXD034030, PXD033493, and PXD050061). The predicted neoantigenic peptide sequences were then searched against this database using the blastp algorithm. Peptides with 100% sequence identity and matching gene annotations were classified as fully matched neoantigenic peptides. For these fully matched peptides, the corresponding protein subcellular localizations were retrieved from the UniProt database to evaluate their membrane association and immunological accessibility.

## Results

### Comprehensive transcriptome analysis revealed the age-related gene in human blood

**FIGURE 1:**
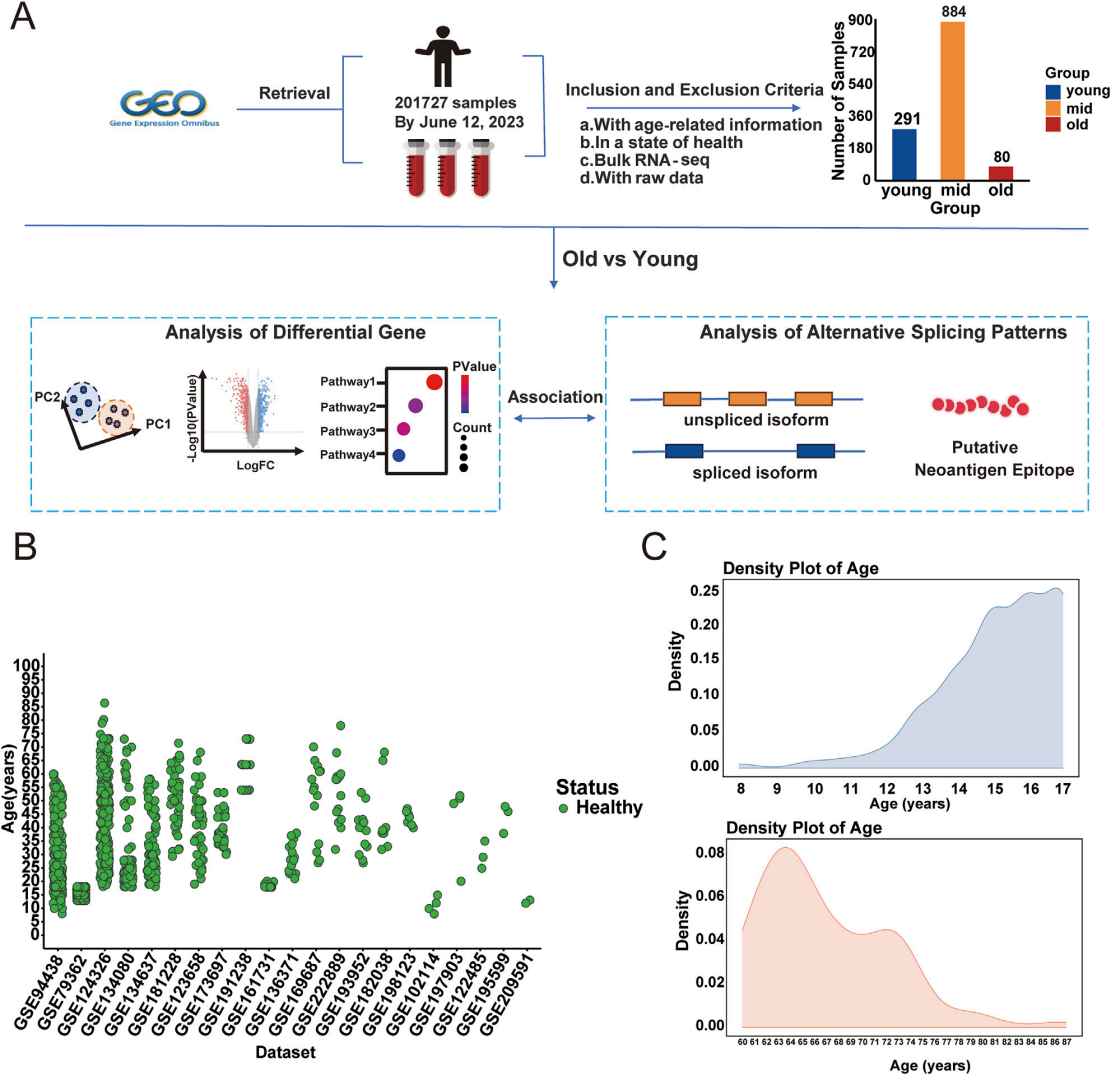
Study design and sample information. **(A)** Overview of sample collection and analytical approach. The samples are categorized into three age groups: young (8–17 years, n = 291), middle-aged (18–59 years, n = 884), and old (60–87 years, n = 80). **(B)** Scatter plot showing the age distribution of samples across the 21 datasets. **(C)** Density plot of age distribution for the young and old groups. The upper panel shows the age density distribution for the young group (The young samples predominantly spanned ages 15–16), while the lower panel illustrates the age density distribution for the old group. (The older samples were mainly between 60–67 years).

**FIGURE 2:**
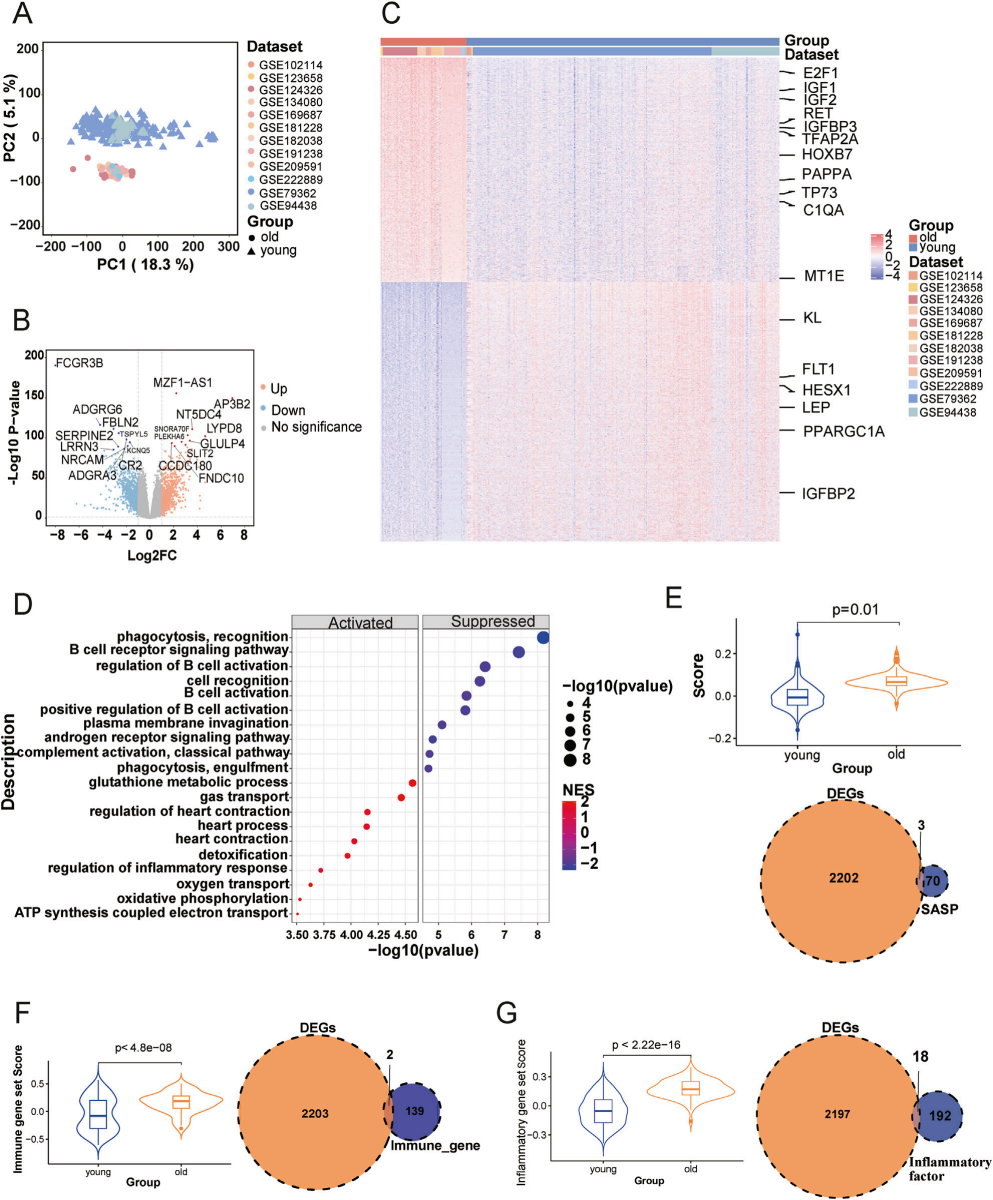
Differential gene expression between young and old groups and its association with immunosenescence. **(A)** Principal component analysis (PCA) of young and old samples. Different datasets are color-coded; circles represent young samples and triangles represent old samples. **(B)** Volcano plot of differentially expressed genes (DEGs) between the young and old groups. Yellow dots indicate upregulated genes (p-value <0.05, Log2-Fold Change ≥1) and blue dots indicate downregulated genes (p-value <0.05, Log2-Fold Change ≤ −1). **(C)** Heatmap showing the expression patterns of DEGs across all samples. Columns correspond to individual samples, annotated by dataset and age group; rows represent DEGs. **(D)** Top 10 upregulated and downregulated biological processes enriched via GSEA in the old group. “Activated” indicates upregulated pathways (p-value <0.05, NES >1), and “Suppressed” indicates downregulated pathways (p-value <0.05, NES < −1). Terms are sorted by ascending p-value. **(E–G)** Immunosenescence characteristics between the two groups. Violin plots display SASP scores (**(E)**, upper), immune scores (**(F)**, left), and inflammatory scores (**(G)**, left) for the young and old groups. Two-tailed t-test p-values are shown. Venn diagrams illustrate the overlaps between dysregulated genes and SASP genes (**(E)**, lower), immune gene sets (**(F)**, right), and inflammatory factors (**(G)**, right).

We collected 21 datasets with RNA-seq data and age information from the Gene Expression Omnibus (GEO), comprising a total of 1,255 healthy human blood transcriptome samples for further analysis. Individuals were categorized into three age groups: young (8–17 years), mid (18–59 years), and old (60–87 years), based on definitions provided by the World Health Organization and prior aging research (Sawyer et al., 2018; Yoshida et al., 2022). Cognitive and memory-related decline has been reported to begin as early as 18 years of age (Chierchia et al., 2023; Salthouse, 2003), while tissue-level aging signals, including neurophysiological and skin structural changes, are detectable at the age of 20 (Salthouse, 2009; Reilly and Lozano, 2021). Moreover, transcriptomic remodeling and systemic molecular aging tend to accelerate around age 60 (Shen et al., 2024; Rando and Wyss-Coray, 2021). Several transcriptomic studies based on peripheral blood have used 60 years old as a threshold to define the aging population (Zhong et al., 2023). To better capture aging-related molecular changes, we focused on the young and old groups, given their larger age span. Figure 1A illustrates the selection, while Figure 1B shows the age distributions across samples. The young samples predominantly spanned ages 15–16, and the older samples were mainly between 60–67 years (Figure 1C). After detecting batch and sex effects between young and old groups, we applied the removeBatchEffect function in limma package. This correction facilitated age-based clustering, thereby improving data integrity (Figure 2A). Differentially expressed genes (DEGs) analysis between young and old groups revealed 1,029 significantly upregulated genes and 1,186 significantly down-regulated genes (p-value <0.05, |Log2FC| > 1; Supplementary Table S4), shown in Figure 2B. Heatmap clustering confirmed the distinct separation of the two groups based on DEGs, demonstrating their biological significance (Figure 2C). To assess the potential impact of sample size imbalance between the young (n = 291) and old (n = 80) groups, we applied a balanced resampling strategy. Specifically, 10 random subsets of 80 young individuals were selected without replacement, and each subset was compared to the old group. As shown in Supplementary Figure S1A, the numbers of up and downregulated genes in these subsets were highly similar to those identified using all young samples, with an average of 1,017 upregulated and 1,166 downregulated genes per subset, compared to 1,029 and 1,186, respectively, in the all comparison. UpSet plots (Supplementary Figure S1B,C) further demonstrated that a majority of the DEGs identified in the all-sample were consistently recovered across the resampled subsets, supporting the stability of our findings.

We further compared these DEGs with 307 known aging-associated genes from the GenAge database (de Magalhães and Toussaint, 2004), identifying 17 overlapping genes, including 11 upregulated and six downregulated genes associated with aging in both human and mouse models (Figure 2C). Notable examples include *TFAP2A*, which is associated with developmental abnormalities and potentially human aging (Schorle et al., 1996); *HOXB7*, which is linked to extended lifespan (Venkataraman and Futerman, 2002); *PAPPA*, whose deletion in mice resulted in a significant 30%–40% increase in lifespan (Conover and Bale, 2007); *TP73*, whose dysregulation increases susceptibility to neurodegenerative diseases (Wilhelm et al., 2010).The *C1QA* gene promotes aging by activating the Wnt signaling pathway, with elevated expression in the brains of aging mice and humans (Naito et al., 2012). Overexpression of *MT1E* in a mouse model resulted in a 14% increase in lifespan (Yang et al., 2006). The *Klotho* gene, which regulates phosphate, calcium, and vitamin D metabolism, is also important; its deficiency in mice leads to premature aging (Donate-Correa et al., 2023; Han et al., 2023). In skeletal muscle, *FLT1* expression decreases with age (Wagatsuma, 2006). The *LEP* gene is involved in aging-related neuroendocrine systems and has been linked to age-related diseases such as diabetes and atherosclerosis (Niu et al., 2023). *PPARGC1A* is associated with cholesterol metabolism, obesity, and age-related diseases such as Parkinson’s disease (Li et al., 2022). The limited overlap between DEGs and known aging-related genes suggests that many aspects of aging mechanisms remain to be understood and warrant further investigation.

Gene set enrichment analysis (GSEA) revealed significant changes in both immunoregulatory and metabolic pathways between young and old groups (Figure 2D; Supplementary Table S5). Specifically, immune pathways like phagocytosis, complement activation, and B cell receptor signaling were downregulated in aging, consistent with age-related immune decline and reduced pathogen clearance. Conversely, pathways related to glutathione metabolism, ATP synthesis, oxidative phosphorylation, and detoxification were upregulated in aging samples, likely representing adaptive responses to mitigate oxidative stress and maintain metabolic functions.

### Age-specific gene expression is not sufficient to capture the intricacies of immunosenescence process

We next explored the association between DEGs and the process of immune senescence. Our analyses revealed significant differences (p ≤ 0.01) in senescence-associated secretory phenotype (SASP), immune, and inflammatory scores between the young and old groups (upper panel of Figure 2E, left panels of Figures 2F,G). SASP, a complex secretome produced by senescent cells that enter a stable arrest state, plays a critical role in recruiting immune cells for their clearance (Birch and Gil, 2020). The elevated immune senescence scores in the older group align with the established characteristics of immunosenescence. However, a limited overlap was observed between DEGs and known SASP, immune, and inflammatory gene sets (lower panel of Figure 2E, right panels of Figures 2F,G). This observation intimates that less-studied genes and molecular pathways may significantly contribute to immunosenescence, beyond the scope of the currently known SASP, immune, and inflammatory genes. These findings highlight the need for further in-depth exploration of immune senescence traits at the molecular level.

### Aging-specific alternative splicing patterns during the aging process

**FIGURE 3:**
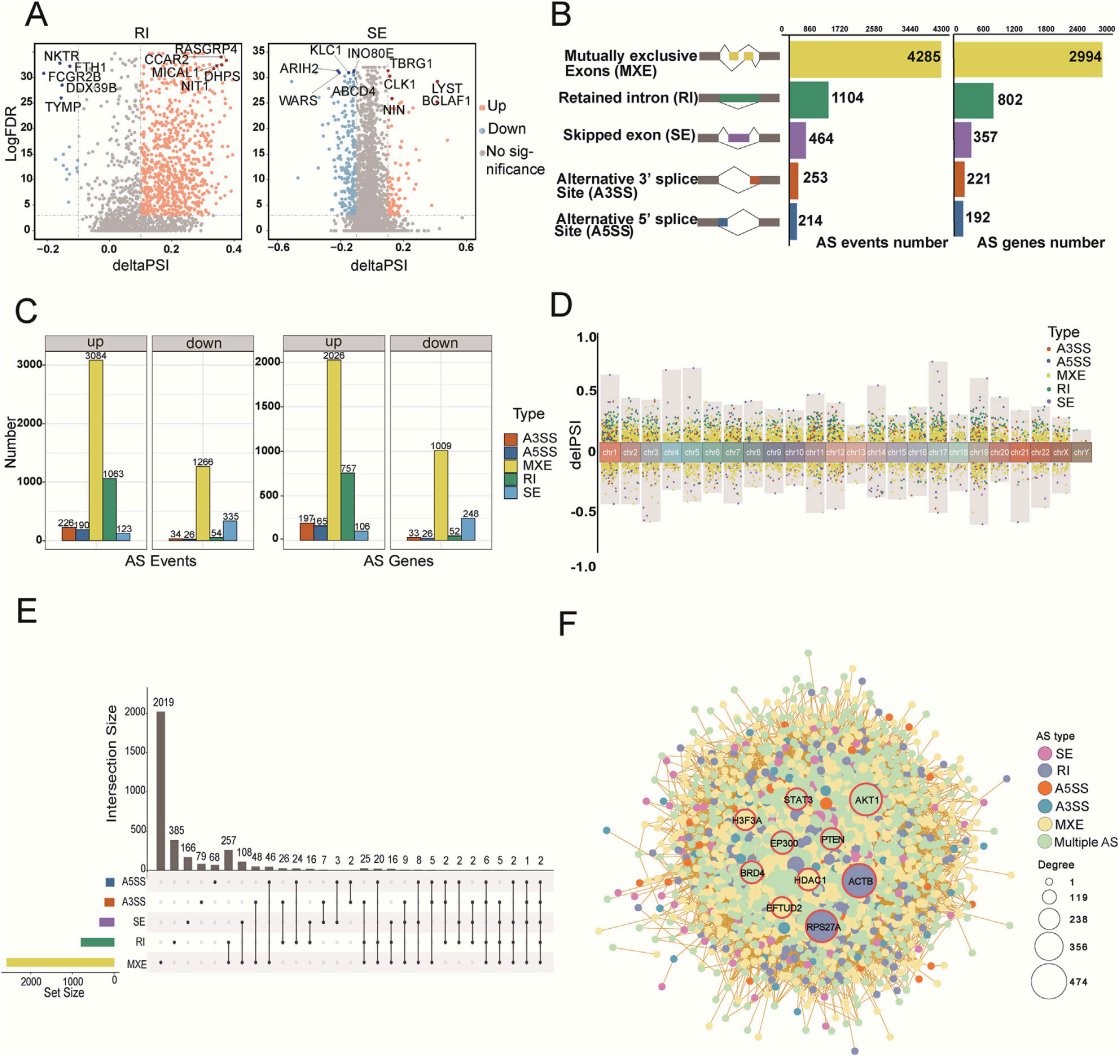
The pattern of alternative splicing (AS) during the aging process. **(A)** Volcano plot showing significant AS events between the young and old groups. Each dot represents a splicing event, with yellow indicating higher usage in the old group (deltaPSI > 0.1), blue indicating lower usage in the old group (deltaPSI < −0.1), and gray indicating non-significant events. The y-axis represents the false discovery rate (FDR). **(B)** Bar plots depicting the counts of significant AS events and their associated genes. The left chart shows the distribution of five types of AS events, the center shows event counts, and the right shows gene counts. **(C)** Facet plots illustrating upregulated and downregulated AS events and their associated genes in the old group. Event counts are shown on the left, and gene counts on the right. **(D)** Chromosomal distribution of significantly different AS events. The upper panel shows upregulated events (deltaPSI > 0.1), while the lower panel shows downregulated events (deltaPSI < −0.1). **(E)** UpSet plot showing the overlap between the five types of AS events detected in the old group. **(F)** Protein-protein interaction (PPI) network analysis of differential splicing events. Nodes represent distinct AS events, with colors indicating the type of splicing event and node size reflecting the degree of connectivity within the network. Edges indicate interactions between nodes. Red circles highlight the top 10 hub genes.

Alternative splicing (AS) is a key regulatory mechanism that drives gene expression diversity and contributes significantly to proteome variation in eukaryotes (Nilsen and Graveley, 2010). Given the limitations of capturing the intricacies of immunosenescence solely at the gene expression level, we quantified differential splicing events during aging to revealits association with immunosenescence. In total, 6,320 splicing events across 4,566 genes were identified using stringent criteria (FDR <0.05, |deltaPSI| > 0.1) (Figure 3A; Supplementary Figures S2A,B; Supplementary Table S6). These included skipped exons (SE), alternative 5′splice sites (A5SS), alternative 3′splice sites (A3SS), mutually exclusive exons (MXE), and retained introns (RI).

The global splicing analysis revealed that MXE (n = 4,285) and RI (n = 1,104) were the most frequent events, whereas SE (n = 464), A3SS (n = 253), and A5SS (n = 214) were less common. MXE and RI events involved the most genes, whereas SE, A3SS, and A5SS events affected fewer genes (Figure 3B). Using the MASER tool, we compared splicing events between the old and young groups. We found that 4,360 splicing events were upregulated in the old group, affecting 3,222 genes. Notably, all splicing event types except SE showed an increasing trend in old samples (Figure 3C). Chromosomal distribution analysis revealed more splicing events on chromosomes 1, 2, and 19, highlighting these regions as transcriptionally active (Figure 3D). Chromosome 1 showed the highest abundance of all five splicing types (Supplementary Figure S2C). We also visualized the overlap of genes involved in different AS types using an upset plot, which showed that more than 14% of genes contained two or more splicing events (Figure 3E). This combination of AS events contributes to the transcriptome complexity and diversity.

AS is critical for generating structural variation in gene products, thereby affecting protein synthesis and function. To understand selective splicing events in the context of protein interaction networks, we constructed protein-protein interaction (PPI) networks using genes with significant splicing events. Ten hub genes were identified, including *ACTB*, *RPS27A*, *AKT1*, *PTEN*, *STAT3*, *BRD4*, *EP300*, *HDAC1*, *EFTUD2*, and *H3F3A*. These hub genes play essential roles in immune regulation and show remarkable variability in splicing during aging. For example, *ACTB* (involved in RI events) influences cytoskeletal maintenance, cell migration, and immune cell function (Gu et al., 2021); *RPS27A* is involved in the cell cycle, immune response, and metabolism, and is closely linked to B- and T-cell antigen recognition (Li et al., 2024); *AKT1* is linked to multiple splicing events, whose aberrant activation contributes to several cancers and may regulate immune cell function (Liu et al., 2014). The PPI maps further demonstrate how splicing influences the overall function of the protein network, highlighting the impact of AS on the molecular mechanism of aging (Figure 3F).

### RNA-binding protein rbms3 as key regulator of aging-related splicing variants

**FIGURE 4:**
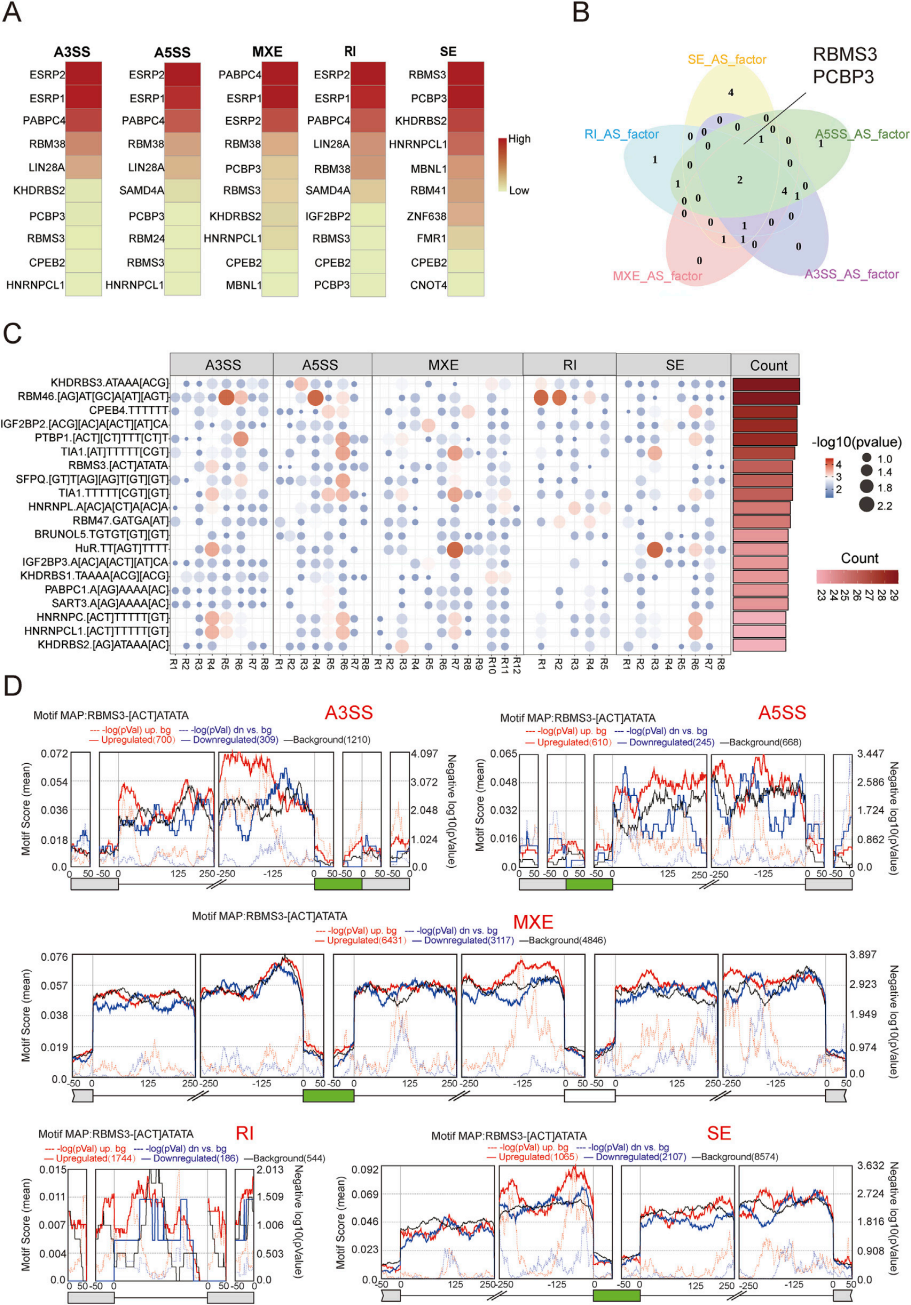
Regulation of alternative splicing (AS) by splicing factors. **(A)** Heatmap displaying the splicing factors associated with the highest number of splicing events across five types of AS events. Darker shades indicate a greater number of associated events. **(B)** Venn diagram showing the overlap of the top 10 splicing factors ranked among the five AS event types. **(C)** Motif enrichment analysis of splicing factors. Each panel corresponds to one type of splicing event (A3SS, A5SS, MXE, SE, and RI). The flanking regions of the splicing events are designated as R1 to R(n). Color intensity and circle size indicate the significance of motif enrichment, with red and larger circles representing the most significant motif binding. The bar chart on the right shows the top 20 regions with the highest motif enrichment significance. **(D)** Distribution of RBMS3 binding motifs across five types of splicing events. Green boxes represent target exons, while gray boxes denote adjacent exons. Lines indicate the intronic sequences 250 base pairs upstream and downstream of each exon. The top panel of each plot shows average motif scores, calculated as the density of nucleotides covered by RBMS3 binding motifs within a 50 base pair sliding window. The black line represents background signal. Solid red and blue lines indicate enrichment of RBMS3 motifs around exons that are more (red) or less (blue) utilized in the old group. Dashed red and blue lines represent the significance of motif enrichment, with higher peaks indicating greater significance.

To further analyze the effects of AS during aging, we investigated the molecular mechanisms driving aging-related splicing variants, specifically the regulatory role of RNA-binding proteins (RBPs) in AS. By analyzing the correlation between the gene expression levels of RBPs and the Percent Spliced In (PSI) values of different splicing event types, we selected the top 10 RBPs with the most correlations for each splicing event type (Figure 4A). Notably, two RBPs (RBMS3 and PCBP3) ranked in the top 10 across all splicing event types (Figure 4B). We then performed enrichment analysis of 115 binding motifs for 91 splicing factors (Supplementary Table S7), observing varying degrees of motif enrichment in regions adjacent to splicing events (Figure 4C). The splicing factors and their corresponding motifs were ranked by the number of significantly enriched regions around splicing events, with the top 20 motifs displayed. Combined with the correlation results in Figure 4A, we found that some splicing factors previously linked to aging were also present in our ranking. For example, HNRNPA1 has been shown to play a central role in aging and tumorigenesis by regulating the 3′untranslated region (3′UTR) length of the HN1 gene, affecting its stability and protein production (Jia et al., 2019). TIA1 is associated with neurodegenerative diseases (Apicco et al., 2018). Of particular interest, RBMS3 ranked within the top 20 and appeared across five splicing event types, suggesting a central role in aging-related splicing variants. Finally, we used rMAPS to visualize the enrichment of the RBMS3 binding motif [ACT]ATATA around splicing event regions. As shown in Figure 4D, RBMS3 binds within 100 base pairs upstream of the target exon, likely promoting exon skipping. Its binding in the downstream intronic region may contribute to the formation of mutually exclusive exon events, while its binding in the upstream exonic region may be involved in intron retention. In addition, RBMS3 binding within the target exon and within 50 base pairs of its 3′end may be associated with to selective 3′splice site events, while its binding in the downstream intronic region may also influence splicing at the 5′splice site.

### Aging-specific alternative splicing is a key regulator in immunosenescence

Immunosenescence is a process of decline in both innate and adaptive immune functions with aging, which contributes to age-related diseases, increased susceptibility to infection, and cancer development. Previous research has highlighted inflammation and T cell status as critical factors in immunosenescence (Liu et al., 2023). Transcriptomic studies of aging T cells have identified numerous alternative splicing events in genes involved in T cell activation, differentiation, migration, and apoptosis. In addition, genes such as *PDCD4* and *ARCN1* have emerged as potential therapeutic targets for mitigating immunosenescence, as they have been implicated in age-specific splicing events and T-cell aging (Mao et al., 2023). To explore the potential link between specific splicing events during aging and immunosenescence, we explored the relationship between AS-related genes, gene expression, and immunosenescence.

**FIGURE 5:**
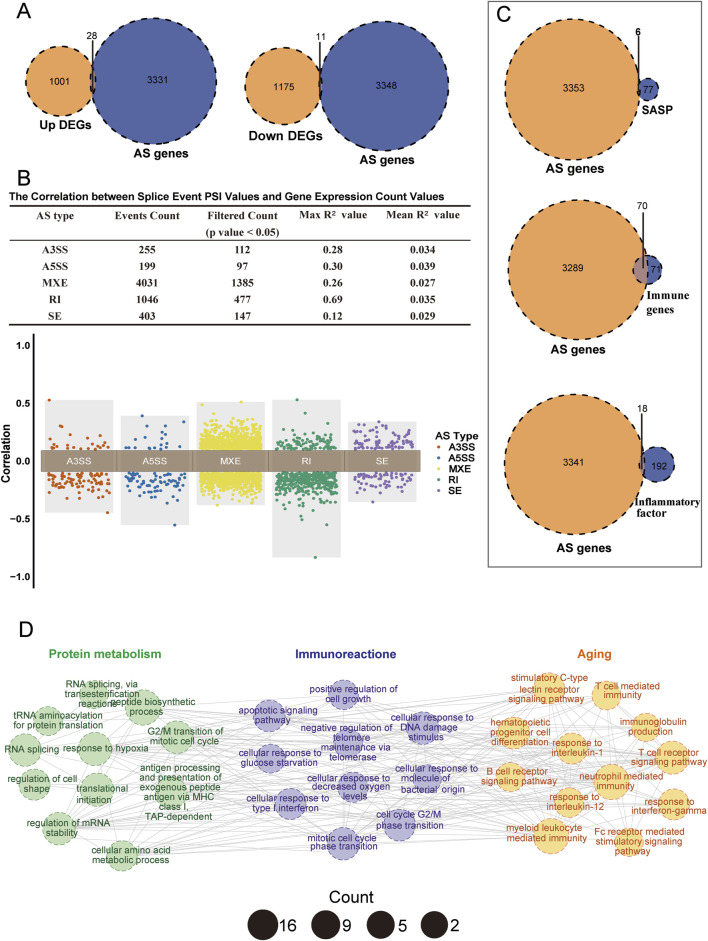
Associations between AS-related genes, gene expression, and immunosenescence. **(A)** Venn diagram showing the overlap of AS-related genes with upregulated genes (left panel) and downregulated genes (right panel). **(B)** Correlation between AS-related genes and gene expression. The upper panel is a table summarizing the correlation between splicing events and corresponding gene expression, including five splicing types (A3SS, A5SS, MXE, SE, RI). It presents the total number of splicing events, the number of significantly correlated events (p < 0.05), the maximum R^2^ value, and the average R^2^ value, where a larger R^2^ indicates a stronger linear relationship. The lower panel is a distribution plot of the correlation coefficients for the five splicing types. Each point represents the correlation coefficient of a splicing event, with different colors representing different splicing types. The y-axis shows positively correlated events (0 < r < 1) and negatively correlated events (−1 < r < 0). **(C)** Venn plots showing the overlap of AS-related genes with SASP (top), immune gene sets (middle), and inflammatory factors (bottom). **(D)** Enrichment network diagram showing significantly enriched pathways (p < 0.05) related to the top 200 splicing events. Similar terms are grouped into larger categories (Protein metabolism, Immune reactions, Aging). Each node represents an enriched term, with size indicating the number of enriched genes, different colors representing different categories, and edges indicating connections between pathways.

We first examined the association between differential splicing events and differential gene expression. Figure 5A shows a small overlap between dysregulated genes and splicing-associated genes, consistent with findings from previous studies (Aktas et al., 2022; Xiao et al., 2024). Specifically, only 28 upregulated and 11 downregulated genes overlapped with AS-associated genes, suggesting limited direct linkage between differentially expressed and AS-associated genes. We further analyzed the correlation between PSI) values of splicing events and gene expression values (Supplementary Table S8). The upper panel of Figure 5B shows that only about 30%–50% of the PSI values had a significant correlation with corresponding gene expression values, with generally low maximum and mean correlation values (Max R^2^, Mean R^2^). These correlations across the five splicing event types mostly clustered around zero (Figure 5B), indicating low correlation between splicing events and gene expression. Only a few retained intron (RI) events showed significant correlation, suggesting that abnormal splicing events do not necessarily lead to noticeable changes in gene expression, and that relying solely on differential gene expression may miss important insights.

Next, we examined the association between differential splicing events and immune senescence. Our analysis revealed that AS-associated genes overlapped more extensively with senescence-associated secretory phenotype (SASP), immune, and inflammatory gene sets than with differentially expressed genes. In particular, there was an increased overlap with immune gene sets (Figure 5C). We selected the top 200 significantly different splicing events for Gene Ontology (GO) analysis, which revealed significant associations with various biological processes (Figure 5D; Supplementary Table S9). These splicing events were enriched in pathways related to protein metabolism, including peptide biosynthesis, RNA splicing, and tRNA aminoacylation—processes essential for intracellular protein homeostasis. Dysregulation of these pathways is strongly associated with aging. Furthermore, these splicing events have been linked to age-related pathways such as the apoptotic signaling pathway and negative regulation of telomere maintenance via telomerase, highlighting telomere shortening as a key marker of cellular senescence (Eppard et al., 2024). In immune-related pathways, differential splicing events were particularly enriched in the T-cell and B-cell receptor pathways, which are critical for adaptive immune responses. The decline of adaptive immunity is a hallmark of immune senescence. Collectively, these findings suggest that differential splicing events are key regulators of protein metabolism, aging, and immune response.

### Aging-related splicing variants disrupt structural domain in immune-related proteins

**FIGURE 6:**
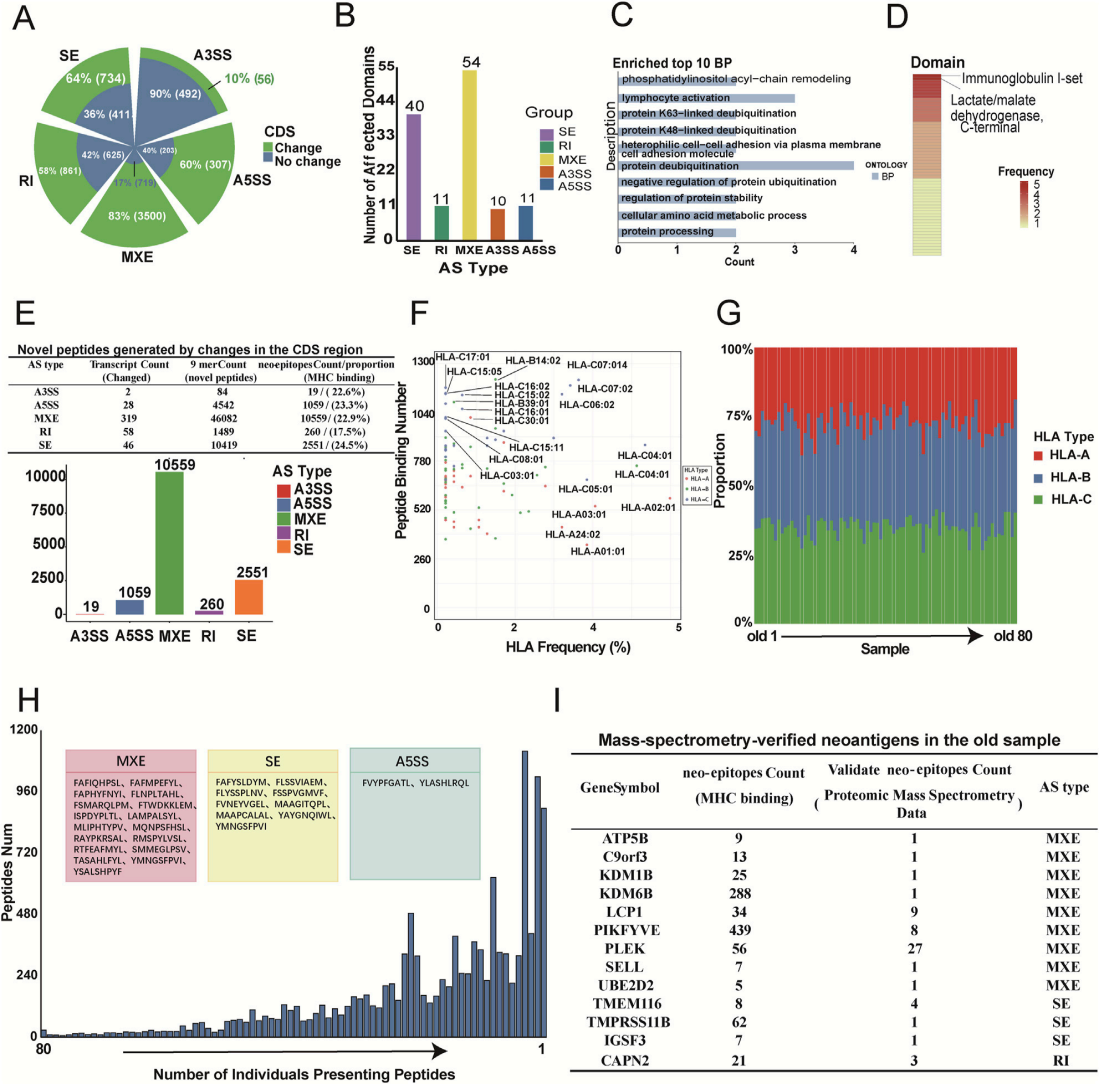
Identification and characterization of aging-associated AS-derived neoantigens. **(A)** Grouped pie chart showing the number and proportion of splicing events with identical (blue) or distinct (green) coding sequences (CDS) across the five types of AS events. **(B)** Bar chart displaying the number of splicing events affecting protein structural domains for each AS type. **(C)** Top 10 biological processes (BP) identified through GO enrichment analysis of genes associated with AS events that affect protein structural domains (p < 0.05). **(D)** Heatmap illustrating the most frequently impacted protein structural domains. Color intensity represents the frequency of impact, with darker shades indicating a greater number of events affecting the same domain. **(E)** Summary of AS-derived peptides and neoantigens. The upper section shows the number and proportion of transcripts altered by the five types of AS events through changes in the CDS, the number of novel peptide segments, and the number of neoantigens binding to MHC molecules. The lower bar chart displays the number of candidate neoantigens generated by the five types of AS events. **(F)** Scatter plot summarizing the frequencies of HLA alleles in elderly individuals and their corresponding counts of bound neoantigens. **(G)** Bar chart showing the number of HLA alleles presenting neoantigenic peptides in 80 elderly samples. Different colors represent different HLA types (HLA-A, HLA-B, HLA-C). **(H)** Bar plot showing the distribution of neoantigenic peptides according to the number of individuals presenting them. The 28 peptides shared across all 80 elderly samples are highlighted in pink (MXE: 17), yellow (SE: 9), and blue (A5SS: 2) boxes. **(I)** Table detailing 60 peptides matched in old blood proteomic data, including gene symbols, peptide counts, verification by mass spectsrometry, and AS types.

Alternative splicing (AS), which occurs in the coding sequence (CDS) regions of genes, leads to changes in protein sequences and disruptions in structural domains. We investigated the effects of AS on protein sequences and structural domains. The results showed that splicing events have a significant impact on protein translation. More than half of the events in splicing types such as retained introns (RI), skipped exons (SE), mutually exclusive exons (MXE), and alternative 5′splice sites (A5SS) affected CDS regions, resulting in changes in protein function (Figure 6A). In particular, MXE and SE events significantly affected several protein structural domains (Figure 6B). Gene ontology (GO) analysis showed that genes affected by these splicing events were enriched in immune-related pathways, including lymphocyte activation, protein deubiquitination, and cellular amino acid metabolism (Figure 6C). These pathways are critical for immune function, and alterations within them may contribute to immune senescence (Damgaard, 2021; Liu et al., 2021). Notably, immunoglobulin class I and the C-terminal domain of lactate/pyruvate dehydrogenase were among the most frequently affected (Figure 6D). Immunoglobulin class I is closely linked to antibody production and immune response (Wang et al., 2020), and its alteration could lead to the formation of new antigens, potentially affecting immune recognition in older individuals. Lactate/pyruvate dehydrogenase plays a vital role in cellular metabolism and energy homeostasis, and its alteration may influence the metabolic state and activity of immune cells (Xiang et al., 2020). These changes in CDS regions and structural domains suggest that significant splicing events not only affect aging-related immune functions, but may also generate new antigens.

### Identification of neoantigens from age-related splicing events as targets for anti-aging immunotherapy

With the rise of immunotherapy, cancer-specific neoantigens have become critical targets for anti-tumor therapies (Huang et al., 2024). Alternative splicing (AS), which regulates gene expression and protein diversity, plays an important role in cancer prognosis. Neoantigens generated by AS could serve as key targets for anti-tumor vaccines or CAR T-cell therapies (Wickland et al., 2024; Du et al., 2024). Although aging and cancer have distinct features, they share commonalities like genomic instability and epigenetic changes. During aging, senescent cells produce more senescence-associated self-peptides (Marin et al., 2023).

This study also explores neoantigens induced by aging-related splicing events. Neoantigen prediction analyses were conducted for significantly upregulated splicing events during aging. Amino acid sequences from spliced and unspliced isoforms were obtained, and these proteins were cleaved into 9-mer peptides. Potential neoantigenic peptides were identified by excluding the peptides normally expressed in adolescent and middle-aged individuals. Using the NetMHCpan-4.1 prediction algorithm, we assessed the likelihood of these new peptides binding to major histocompatibility complex (MHC) molecules (Figure 6E and Methods). The results showed that transcripts with CDS alterations across five splicing types generated numerous neo-peptides, with about a quarter showing strong MHC binding affinity, suggesting that they could serve as candidate neoantigens in aging.

Population-level analysis of HLA alleles revealed that some alleles presented more neoantigenic peptides than others. HLA-C14:02 presented the highest number of neoantigenic fragments (1,254), with a detection rate of 1.9%, while HLA-A02:01 was the most commonly detected allele, present in 4.9% of older samples and presenting 532 neoantigenic peptides (Figure 6F). These findings suggest the feasibility of anti-aging vaccine development based on these peptides. In addition, neoantigenic peptides in aged samples were more likely to be presented by the HLA-B allele (Figure 6G), suggesting that HLA-B may be a preferred target for anti-aging vaccines. Further analysis identified 28 neoantigenic peptides present in all elderly samples (Figure 6H), and 60 potential neoantigenic peptides that validated by mass spectrometry-based proteomics data from elderly individuals (Figure 6I; Supplementary Table S10). Notably, peptides produced through MXE splicing were associated with the senescence-related gene *UBE2D2*, which encodes a ubiquitin-binding enzyme essential for maintaining protein homeostasis and delaying aging (Hunt et al., 2024). These findings support the aberrant production of neoantigenic peptides in the elderly population and highlight the potential for immunotherapy targeting these peptides in anti-aging treatments.

## Discussion

The biological process of aging involves genomic instability, telomere attrition, epigenetic modifications, and disruptions in protein homeostasis (López-Otín et al., 2023). These changes compromise physiological integrity over time, leading to organ dysfunction and an increased risk of age-related diseases, such as cancer and cardiovascular conditions. While current therapies target these complications, their effectiveness in extending a healthy lifespan is limited. The immune system also undergoes significant age-related changes, such as altered immune cell composition, increased inflammation, and functional decline (Mogilenko et al., 2022). Despite these insights, identifying effective targets for anti-aging immunotherapy remains a challenge, necessitating a deeper understanding of the aging mechanisms.

In this study, transcriptomic analysis was conducted on blood samples from individuals across various age groups to investigate changes in gene expression and alternative splicing patterns associated with aging. Data from 1,255 healthy samples obtained from the GEO database were categorized into young, middle-aged, and old groups (ages 8–87 years), based on developmental and molecular aging patterns supported by previous studies. Given our study’s focus on aging-specific alternative splicing (AS), a groupwise comparison between biologically distant age extremes offers greater sensitivity. Previous work has demonstrated that AS changes are more evident at developmental boundaries than during midlife (Kim et al., 2025). In addition, our splicing analysis tool, rMATS, supports only binary group comparisons and does not accommodate continuous age modeling. PSI values were also unavailable for middle-aged individuals, limiting the feasibility of regression analysis. Including this large middle-aged cohort in either group would introduce imbalance and confound interpretability. Therefore, we focused on young vs. old comparisons for transcriptomic and splicing analyses, and utilized the middle-aged cohort *post hoc* to filter splicing noise and minimize false-positive neoantigen identification. Although the young and old groups differ in sample size, our balanced resampling analysis (10 iterations of randomly selecting 80 young individuals) produced comparable results to those obtained using all young samples, both in terms of the number of differentially expressed genes and the identification of shared genes. These findings suggest that the observed transcriptomic differences between age groups are not merely artifacts of sampling imbalance, but likely reflect genuine biological variation. To begin, we compared the transcriptomes of the young and old groups, and the results showed that only a few of the differentially expressed genes overlapped with known aging, senescence-associated secretory phenotype (SASP), immune, and inflammatory gene sets. Gene set enrichment analysis (GSEA) indicated downregulation of immune-related pathways, such as phagocytosis and complement activation, and upregulation of pathways linked to antioxidant metabolism, energy production, and oxygen transport during aging. These findings align with those of Lorna W. Harries et al., who observed a small number of age-related transcripts using transcriptome microarray technology on human blood leukocytes from individuals aged 30–104 years (Harries et al., 2011). In their study, only 295 out of 16,571 transcripts (∼2%) were strongly associated with aging, including several linked to inflammation and immune senescence. Although our dataset represents the most comprehensive RNA sequencing data available for healthy older individuals in the GEO database as of June 2023, the smaller sample size in the older age group may impact statistical robustness. Including more elderly samples in future studies would improve the depth and reliability of these findings. Moreover, Harries et al. highlighted the role of mRNA processing in aging, providing valuable insights for exploring alternative splicing in our study. Our RNA sequencing approach offers advantages over microarray technology, particularly for analyzing splicing isoforms and alternative splicing events.

Current research into aging mechanisms has expanded from DNA damage and protein homeostasis to RNA processing. In particular, mRNA splicing, which plays a crucial role in regulating gene expression and generating protein diversity, has been closely linked to aging and longevity interventions (Bhadra et al., 2020; Baralle and Romano, 2023). Holly et al. reported increased alternative splicing events in senescent fibroblasts and endothelial cells (Holly et al., 2013). Similarly, Stegeman et al. identified variable splicing changes in *Drosophila*, underscoring the conserved role of splicing in senescence across species (Stegeman et al., 2018). In our study, variable splicing patterns in human blood increased with age, and retention of introns (RI) significantly rose, consistent with aging signatures identified by Adusumalli et al. (Adusumalli et al., 2019). The choice of alternative splicing analysis tool can affect the interpretation of results. The rMATS tool, which uses predefined splicing models and compares splicing patterns between sample sets, is more efficient and accurate than tools like LeafCutter for large datasets (Aktas et al., 2022). We used the rMATS tool, identifying 6,320 splicing events associated with aging across 4,566 genes. To address concerns about sample imbalance, we applied a single balanced resampling step by selecting 80 young individuals and comparing them to the old group, due to the computational demands and BAM-format input required by rMATS. As shown in Supplementary Figure S3A, the number of genes involved in significantly altered AS events—except for SE—was higher in old individuals, consistent with results from the all sample. Additionally, Supplementary Figure S3B shows a substantial overlap between AS-related genes identified in the resampled subset and those from the all comparison, although some differences remained. These differences may reflect the increased sensitivity of AS detection to sample size, noise, and methodological constraints. Nonetheless, the similarity in trends supports the validity of using all samples to capture aging-associated splicing alterations. The 4,566 genes affected by splicing alterations included many that overlapped with known aging, SASP, immune, and inflammatory gene sets. And these genes are involved in regulating protein metabolism, immune responses, and signaling pathways, suggesting that splicing alterations could be valuable targets for anti-aging immunotherapy. Dysregulated pre-mRNA splicing can lead to age-associated pathological conditions, and fine-tuning the regulation of splicing factors could delay aging and treat age-related diseases (Angarola and Anczuków, 2021). For example, splicing factors such as HNRNPA1 and TIA1 are strongly associated with aging and aging-related diseases (Jia et al., 2019; Apicco et al., 2018), and both ranked highly in our splicing factor correlation results. Notably, RBMS3 was found to be highly correlated with splicing events across all five splicing types, suggesting that its dysregulation may play an important role in the splicing patterns of aging. RBMS3 belongs to the MSSP family of RNA-binding proteins and has been characterized as a tumor suppressor gene involved in apoptosis, post-transcriptional mRNA regulation, and fibrosis. Tumorigenesis is an age-associated process, and reduced RBMS3 expression has been linked to poor prognosis in various cancers (Li Wen et al., 2023). Recent studies have also suggested that RBMS3 may be involved in immune regulation and aging. Specifically, RBMS3 suppression was found to downregulate PD-L1 and enhance immune responses (Zhou et al., 2023), while metformin treatment increased RBMS3 expression and promoted ferroptosis in ovarian cancer cells (Zhao et al., 2025). As metformin has been shown to reset aging clocks and delay aging in primates (Yang et al., 2024), these findings raise the possibility that RBMS3 could participate in the regulation of immunosenescence and age-associated cellular remodeling. These findings highlight a strong link between changes in splicing patterns during aging and immune senescence, which may lead to the identification of new targets for anti-aging immunotherapy.

In cancer therapy, personalized vaccines targeting neoantigens can be developed by predicting variable splicing mutations in tumor cells, stimulating a specific immune response against the tumors (Lybaert et al., 2023; Xie et al., 2023). Given the similarities between cancer and aging, the discovery of age-specific neoantigens has become a major research focus. Vaccines have already entered clinical trials for age-related diseases such as Alzheimer’s disease (Wu et al., 2024; Hovakimyan et al., 2024). Amor et al. analyzed the transcriptome of senescent cells and identified proteins widely expressed and specifically anchored to cell membranes, which can serve as CAR T cell targets for the removal of senescent cells (Amor et al., 2024). Following the workflow of Rui Cheng et al. (Cheng et al., 2021), we identified new epitopes generated by specific variable splicing during aging and validated 60 neoantigenic peptides using mass spectrometry-based proteomics data from elderly individuals. Notably, several of these peptides are derived from membrane-associated proteins, suggesting their potential accessibility to immune surveillance. However, due to the limitations of sequence-based analyses, these findings require further experimental validation to assess their immunogenicity and therapeutic applicability. Future studies will focus on experimentally confirming the surface presentation of these neoantigens and evaluating their potential as targets for anti-aging immunotherapies.

Despite providing valuable insights, our study has several limitations. Although we accounted for sex as a confounding factor—an adjustment that showed minimal impact on differential expression outcomes in our dataset (Supplementary Figures S4A,B)—other demographic and environmental variables such as ethnicity, comorbidities, medication use, diet, and socioeconomic status may also influence gene expression and splicing profiles. We incorporated all available clinical metadata from the public datasets used; however, detailed annotations were often incomplete. This lack of comprehensive metadata may limit the generalizability and interpretability of our findings. Future studies incorporating more comprehensive sample-level annotations could better account for the influence of additional confounding variables on age-related transcriptomic changes. Moreover, While neoantigens derived from aging-associated splicing events present promising therapeutic opportunities, their use in immunotherapy must be approached cautiously (Huang et al., 2024). Aging tissues may express peptides that were not encountered during thymic development, potentially bypassing central tolerance (Xie et al., 2023) and raising the risk of autoimmunity (Mustelin and Andrade, 2024). The aging immune system exhibits altered peripheral tolerance and a pro-inflammatory environment, which could exacerbate immune-related side effects. Similar risks have been observed in cancer CAR-T therapies, where even low-level antigen expression in normal tissues can result in severe toxicity (Sebastian-Valverde and Pasinetti, 2020). Strategies such as stringent target selection (e.g., membrane-bound proteins highly enriched in senescent cells) (Flugel et al., 2023), combinatorial antigen gating, and built-in safety switches may help mitigate these effects (Huang and Liu, 2020). Nevertheless, the use of CAR-T cells or other immune strategies against aging-related neoantigens should be regarded as exploratory, requiring thorough preclinical validation to ensure specificity, safety, and immune tolerance (Feucht and Abou-El-Enein, 2020).

In summary, this study investigated age-related changes in gene expression and their association with immune system decline using blood transcriptome analysis. We uncovered distinct patterns of alternative splicing associated with human aging and identified novel antigens produced by abnormal splicing events that may serve as promising targets for anti-aging immunotherapy. Our study provides new insights into alternative splicing during aging, highlights promising avenues for anti-aging immunotherapy, and advances our understanding of immunosenescence.

## Data Availability

The raw data and the analysis codes are available in the Supplementary Material and made publicly available at GitHub repository via https://github.com/lsh74/ASNPB.
