# Supplementary material for: Aging-related alternative splicing drive neoantigen emergence revealed by transcriptome analysis of 1,255 human blood samples
Source: Front Aging. 2025 May 9;6:1575862. doi: 10.3389/fragi.2025.1575862 (PMC12098113; doi:10.3389/fragi.2025.1575862)
Supplement: Supplementary file 1 [file DataSheet1.zip › Supplementary_Figures_and_Legends.docx]

**Supplementary Figures and Legends**

**Figure S1**

**
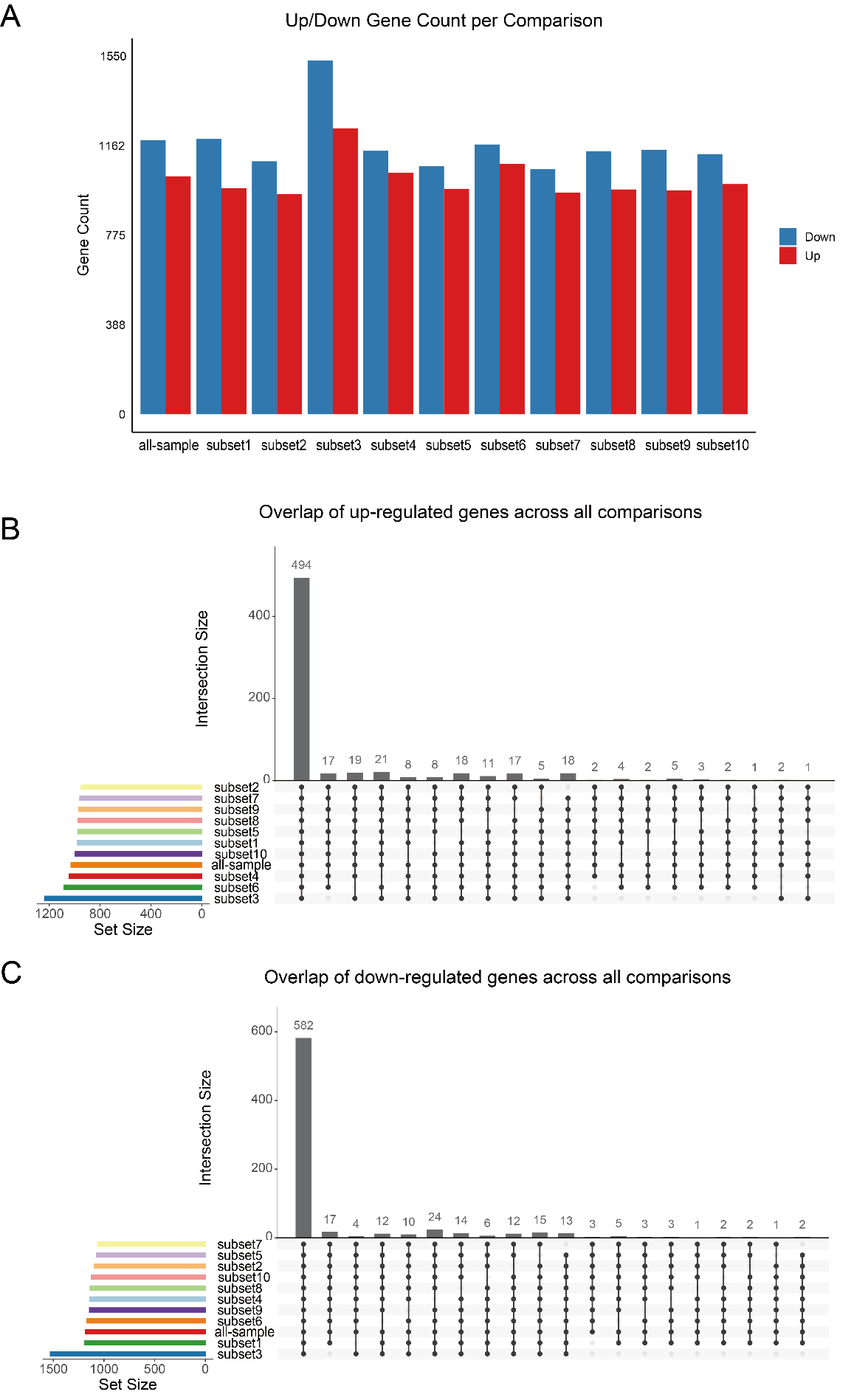
**

**Figure S1**. **Balanced resampling strategy for differential expression analysis.**

(A) Barplot showing the number of up- and down-regulated genes in each comparison (10 10 subsets vs. full-sample comparison). (B) UpSet plot showing the overlap of up-regulated genes across all comparisons (10 subsets + full-sample). (C) UpSet plot showing the overlap of down-regulated genes across all comparisons (10 subsets + full-sample).

**Figure S2**


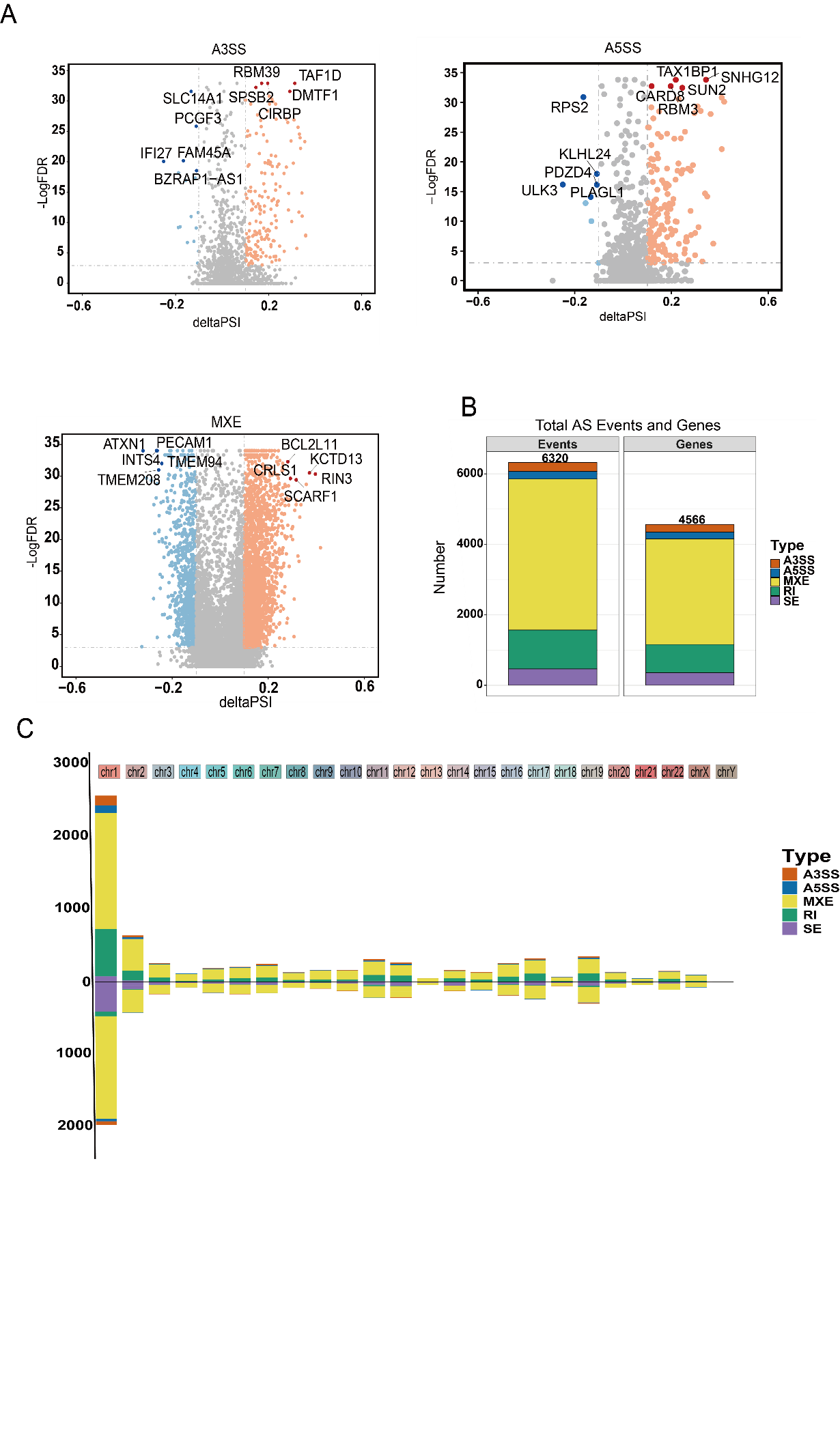
**Figure S2. The pattern of alternative splicing (AS) during the aging process.**

(A) Volcano plots for the three types of events: Alternative 3' Splice Site (A3SS), Alternative 5' Splice Site (A5SS), and Mutually Exclusive Exons (MXE). (B) Bar chart displaying the number of splicing events and genes. (C) Distribution of splicing event counts across different chromosomes.

**Figure S3**

**
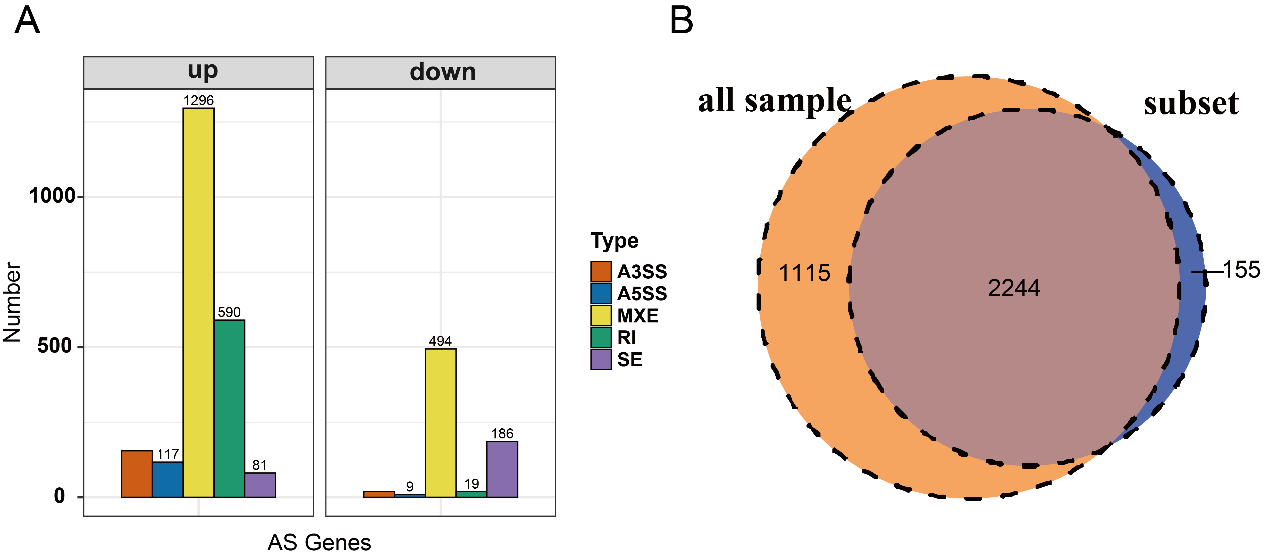
**

**Figure S3. Aging-related AS analysis based on a balanced resampling strategy.**

(A) Numbers of up- and down-regulated AS**-**related genes by splicing type in the resampled comparison. Most event types showed more genes in the old group, consistent with all-sample results. (B) Overlap of AS-related genes between the all sample and the resampled subset.

**Figure S4**

**
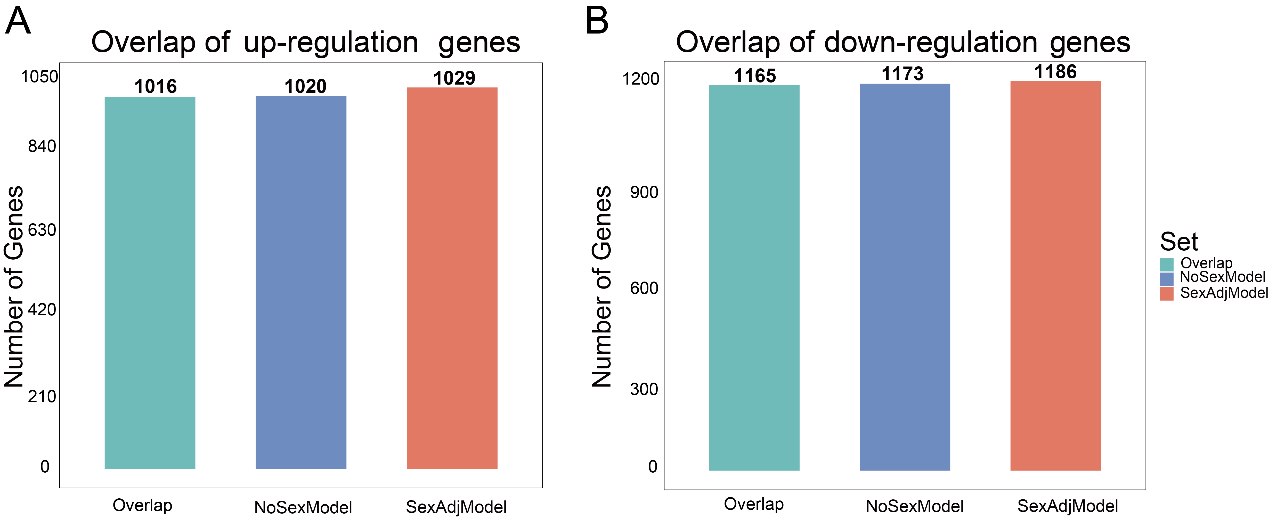
**

**Figure S4. Effect of sex adjustment on differential gene expression analysis between young and old groups*.***

(A) Overlap of upregulated genes identified with and without sex adjustment. (B) Overlap of downregulated genes identified with and without sex adjustment.
